# Genetics of response to cognitive behavior therapy in adults with major depression: a preliminary report

**DOI:** 10.1038/s41380-018-0289-9

**Published:** 2018-11-08

**Authors:** Evelyn Andersson, James J. Crowley, Nils Lindefors, Brjánn Ljótsson, Erik Hedman-Lagerlöf, Julia Boberg, Samir El Alaoui, Robert Karlsson, Yi Lu, Manuel Mattheisen, Anna K. Kähler, Cecilia Svanborg, David Mataix-Cols, Simon Mattsson, Erik Forsell, Viktor Kaldo, Martin Schalling, Catharina Lavebratt, Patrick F. Sullivan, Christian Rück

**Affiliations:** 1 Centre for Psychiatry Research, Department of Clinical Neuroscience, Karolinska Institutet, Stockholm, Sweden; 2 Stockholm Health Care Services, Stockholm County Council, Stockholm, Sweden; 3 Center for Psychiatric Genomics, University of North Carolina, Chapel Hill, NC USA; 4 Department of Genetics, University of North Carolina, Chapel Hill, NC USA; 5 Division of Psychology, Department of Clinical Neuroscience, Karolinska Institutet, Stockholm, Sweden; 6 Department of Medical Epidemiology and Biostatistics, Karolinska Institutet, Stockholm, Sweden; 7 Department of Biomedicine and Center for Integrated Sequencing (iSEQ), Aarhus University, Aarhus, Denmark; 8 Department of Psychology, Faculty of Health and Life Sciences, Linnaeus University, Växjö, Sweden; 9 Neurogenetics Unit, Department of Molecular Medicine and Surgery, Karolinska Institutet, Stockholm, Sweden; Center for Molecular Medicine, Karolinska University Hospital Solna, Stockholm, Sweden

**Keywords:** Predictive markers, Psychology

## Abstract

Major depressive disorder is heritable and a leading cause of disability. Cognitive behavior therapy is an effective treatment for major depression. By quantifying genetic risk scores based on common genetic variants, the aim of this report was to explore the utility of psychiatric and cognitive trait genetic risk scores, for predicting the response of 894 adults with major depressive disorder to cognitive behavior therapy. The participants were recruited in a psychiatric setting, and the primary outcome score was measured using the Montgomery Åsberg Depression Rating Scale-Self Rated. Single-nucleotide polymorphism genotyping arrays were used to calculate the genomic risk scores based on large genetic studies of six phenotypes: major depressive disorder, bipolar disorder, attention-deficit/hyperactivity disorder, autism spectrum disorder, intelligence, and educational attainment. Linear mixed-effect models were used to test the relationships between the six genetic risk scores and cognitive behavior therapy outcome. Our analyses yielded one significant interaction effect (B = 0.09, *p* < 0.001): the autism spectrum disorder genetic risk score correlated with Montgomery Åsberg Depression Rating Scale-Self Rated changes during treatment, and the higher the autism spectrum disorder genetic load, the less the depressive symptoms decreased over time. The genetic risk scores for the other psychiatric and cognitive traits were not related to depressive symptom severity or change over time. Our preliminary results indicated, as expected, that the genomics of the response of patients with major depression to cognitive behavior therapy were complex and that future efforts should aim to maximize sample size and limit subject heterogeneity in order to gain a better understanding of the use of genetic risk factors to predict treatment outcome.

## Introduction

Major depressive disorder (MDD) is a common and heritable illness with a lifetime prevalence of around 14–15% [1, 2]. Twin studies have demonstrated that ~40% of the variation in the liability to MDD is attributed to additive genetic effects [3]. However, the genetic foundations of MDD have long been unclear [4], although a recent genome-wide association study (GWAS) that included 130,664 cases reported 44 loci associated with MDD [5]. Cognitive behavior therapy (CBT), which is effective for MDD, is considered the treatment of choice for mild-to-moderate MDD according to international guidelines [6, 7]. Unfortunately, access to CBT is limited to many patients due to cost, lack of trained therapists, and geographical barriers [8]. Internet-delivered CBT (iCBT) is an online form of therapist-guided CBT that is presented as a series of modules accompanied by homework assignments over multiple weeks [9]. iCBT has shown effect sizes comparable with traditional face- to-face CBT [10], and it may help bridge the supply and demand gap [11–15]. In addition, iCBT has the advantage of providing rich phenotypic information due to its highly monitored and manualized treatment protocol that often makes the progression of treatment contingent on filling out forms at different time points [16]. However, approximately half of the patients who undergo CBT do not respond [13]. Therefore, an important step toward identifying those who are more likely to respond to treatment is to distinguish the predictors of outcome to prevent patients from experiencing treatment failure.

Several studies have suggested clinical predictors of CBT outcome, such as baseline symptom severity [17], psychopathological comorbidity [18], having a social support network [19, 20], greater burden of illness [21–23], treatment adherence [24], and working full-time employment [25].

However, the results have been mixed [17]. Therefore, factors with acceptable predictive power to guide clinical decisions are not currently available [26].

The study of genetic predictors of psychological treatment outcome, sometimes called therapygenetics [27], is a fairly new field [28]. Similar to pharmacogenetics, the aim of therapygenetics is to use genetic data to better predict the outcome of psychological treatment and personalize interventions [29]. The earliest work in the field of therapygenetics has focused on candidate genes [27], but many of the findings were not replicable or inconsistent [30, 31], Because the effects of each genetic factor that influences therapy response are likely small and dispersed across the genome, these types of analyses should focus on genome-wide variations rather than single polymorphisms. The first GWAS of CBT response, of 980 subjects with anxiety disorders did not detect any significant common variants [32]. However, an epigenetic study reported an association of monoamine oxidase A methylation with CBT response in individuals with panic disorder [33]. In addition, the results of a subsample in a recent genome-wide expression study suggested the association of a few genetic variants with exposure-based treatment response in 102 patients with panic disorder and specific phobias [34].

However, experience from pharmacogenetic studies implicates the unlikeliness of finding strongly replicated single-nucleotide polymorphisms (SNPs) with a large contribution to a complex trait, such as treatment response, and that large samples and well-defined homogeneous phenotypes are needed [35]. Attempts have therefore been made to aggregate the effects of common genetic variants to identify or explain a meaningful proportion of the genetic load of treatment response by calculating genetic risk score (GRS) [36], which quantifies the inherited burden of common variants across the genome for a given *p*-value threshold.

The present study explored the utility of GRSs for predicting treatment response in a sample of 894 subjects with MDD who underwent a standardized iCBT protocol. We tested the hypothesis that a GRS for MDD and five other psychiatric (bipolar disorder, ADHD and autism spectrum disorder) and cognitive traits (intelligence and educational attainment) are associated with the effects of iCBT treatment over time. We chose these traits because cause often informs cure [37], greater genetic risk for psychopathology could index severity that is suggested a predictor of poor outcome [38], and cognitive ability that has been associated with CBT treatment response [39]. To the best of our knowledge, this is the first study to fully investigate the genetic risks of psychological treatment response in MDD.

## Materials and methods

### Subject characteristics

Between 2008 and 2016, adult patients with MDD who started iCBT at the Internet Psychiatry Clinic in Stockholm [40], a government-funded psychiatric clinic specializing in delivering psychologist-guided iCBT, were asked to participate in the study. The treatment center is part of the public psychiatric care provided by the Stockholm County Council. The patients were asked to donate a blood sample for DNA. The patients had either been referred to the clinic by their general practitioner or via an online self-referral system. See Table 1 for a full description of the 894 study participants included in the final analysis. As detailed below, individuals from the original sample of 964 were excluded from the study for the following reasons: being an ancestry outlier (*n* = 49), quality control issues (*n* = 11), and missing phenotypes (*n* = 10).

**Table 1:** Demographic characteristics of the participants

Variable	Sample^1^ *N* = 894
*Gender*	
Women	586 (65.5%)
Men	308 (34.5%)
*Age*	
Mean age (SD)	37.9 (11.8)
Range	18–75
*Occupational status*	
Working	640 (71.6%)
*Education*	
7–9 years in school	16 (1.8%)
Incomplete vocational or secondary school	35 (3.9%)
Vocational school	53 (5.9%)
Secondary school	183 (20.5%)
University/college, uncompleted	168 (18.8%)
University/college, completed	437 (48.9%)
Other or unknown	2 (0.2%)
*Relationship status*	
Married or de facto	493 (55.1%)
Single	261 (29.2%)
Divorced	135 (15.1%)
Widow/widower	3 (0.3%)
Missing	2 (0.2%)
*Psychotropic medication*	
Previously (yes)	372 (41.6%)
Currently (yes)	514 (57.5%)
*Comorbidity*	
Comorbid anxiety disorder	202 (22.6%)
Comorbid other	18 (0.2%)
*MDD type at inclusion*	
Mild	134 (15.0%)
Moderate	193 (21.6%)
Severe	2 (0.2%)
Recurrent mild	170 (19.0%)
Recurrent moderate	316 (35.3%)
Recurrent severe	9 (1.0%)
Other	70 (8.0%)
*Suicide attempts*	
Previously (yes)	52 (5.8%)

*SD* standard deviation, *MDD* major depressive disorder

^1^All values are *n* (% of total) unless otherwise noted

After an online screening, the patients came to the clinic for psychiatric assessments, including a structured diagnostic interview (Mini-International Neuropsychiatric Interview) [41]. A psychiatrist or supervised psychiatry resident performed the interview. For enrollment in the study, the patient had to meet the following requirements: fulfill the criteria in the DSM IV-TR for current MDD [42, 43], be able to read and write in Swedish, and be at least 18-year-old. The exclusion criteria were any of the following: severe MDD combined with moderate to high risk of suicide, recent medication changes, comorbid bipolar or other psychotic disorder, unable to participate in concurrent psychotherapy, current alcohol or illicit drug abuse/dependence, or communication difficulties that impact treatment. The study was approved by the Regional Ethics Board in Stockholm, Sweden. All participants provided written informed consent.

### Intervention

The core interventions of iCBT are the same as those administered face-to-face in conventional CBT. The iCBT program consisted of 10 text modules with components covering standard CBT interventions for patients with MDD, such as psychoeducation, cognitive restructuring, behavioral activation, and relapse prevention, that were to be completed in 12 weeks. Each module had a set of tasks and homework assignments to be completed each week that were monitored by the therapist via the secure online platform. In general, the patient and therapist interactions were limited to email contact, and there were no live meetings. A thorough description of the program has been published previously [44].

### Primary outcome measure

The primary outcome measure was assessed using the Montgomery Åsberg Depression Rating Scale-Self report (MADRS-S) [45]. The MADRS-S total score, which ranges from 0 to 54, measures nine clinical characteristics of depression. The MADRS-S was assessed at treatment start (MADRS-S baseline), once each week during treatment, and in the last week of treatment (MADRS-S Post). Thus, each individual provided up to 12 weekly MADRS-S assessments that were included in the analyses. See Supplementary Table 2.

### Genotyping

Genotyping was performed at LIFE & BRAIN GmbH (Bonn, Germany) using the Infinium Global Screening Array 1.0 BeadArray (Illumina, Inc., San Diego, CA, USA) and automated workflow according to the manufacturer's instructions. The raw data were analyzed using GenomeStudio 2.0 (Illumina, Inc.) using the Infinium cluster file (GSA-24v1-0_A1_ClusterFile.egt). A reclustering step was performed using the GenTrain 3 algorithm in Genome Studio 2.0.

### Discovery datasets

GRSs were generated for the following six phenotypes: MDD, bipolar disorder (BIP), attention-deficit/hyperactivity disorder (ADHD), autism spectrum disorder (ASD), intelligence (IQ), and educational attainment (EDU). We obtained the corresponding GWAS results for MDD, BIP [46], ADHD [47], and ASD [48] from the Psychiatric Genomics Consortium (PGC) website (https://www.med.unc.edu/pgc/results-and-downloads) and the GWAS results for IQ and EDU from published GWAS meta-analyses [49, 50]. The target set (currently studied iCBT samples) were not part of these previous GWAS meta-analyses.

### Target dataset

The GWAS data from the 964 iCBT samples were processed using the PGC Ricopili pipeline for quality control and genotype imputation with reference genomes from the 1000 Genomes Project (phase 1 version 3) [51]. Eleven samples were excluded due to sample overlap (two pairs), cryptic relatedness (two pairs with pi-hat ≥ 0.2), or poor call rate (three samples). After excluding 49 subjects due to non-European ancestry, the top 20 ancestry principal components (PC) were calculated from the best-guess imputed genotypes, please see Supplementary Figure 1. Ten participants who failed to start treatment after inclusion were excluded due to missing phenotype data, resulting in a final sample total of 894. The details of the SNP quality control of the discovery and target datasets and reference data, together with the overlapping numbers of SNPs among these three sets, are provided in Supplementary Figure 2.

### GRS calculation

The GRS values were derived for the target set iCBT samples as the sum of the scores based on the risk alleles weighted by the effect size from the discovery sample. To select an independent set of SNPs for calculating the GRS, we conducted linkage disequilibrium clumping (r^2^ < 0.1 in 1-Mb window) on the overlapping SNPs using the European samples from the 1000 Genomes Project as a linkage disequilibrium reference. We computed eight sets of GRS for each phenotype under the *p*-value cutoffs of ≤ 1x10^-5^, ≤ 1x10^-4^, ≤ 0.001, ≤ 0.01, ≤ 0.05, ≤ 0.1, ≤ 0.5, ≤ 1. The GRS calculations were performed using PLINK (version 1.9) [52].

### Statistical analyses

The statistical analyses were performed using *R* [53]. To analyze the association between the six calculated GRS values and iCBT treatment outcome measured by MADRS-S, we used the lme4 package [54] to perform full information maximum likelihood mixed models, including all available data for all patients. First, we fitted a model that determined the overall course of the MADRS-S values over the treatment period. This model included linear and quadratic effects of time (to allow for curvilinear development over time, which provided the best fit of the data) as fixed effects. The model also included a random intercept and random effect of time. Second, we investigated the influence of GRS on the rate of change during treatment. In all models, covariates (i.e., GRS) and possible confounders (i.e., ancestry PC scores, age, and sex) were added as both main effects and interaction effects with linear effect of time. The interpretation of a significant main effect of a GRS is that the GRS had a constant effect on the MADRS-S rating throughout the entire treatment period. The interpretation of a significant GRS × time interaction effect is that the GRS influenced the rate of improvement during treatment. These analyses were performed in the following steps: (1) Each of the six GRS domains at the predetermined *p*-value cutoff were investigated in separate models while controlling for the top five ancestry PC scores. (2) Age and sex were added to the models in step 1. (3) A full model was created in which all six GRSs were entered while controlling for ancestry PCs, age, and sex. As stated above, all covariates (GRS scores, ancestry PCs, age, and sex) were entered as both main effects and interaction effects with linear time in these analyses. To reduce multiple testing, we tested each of the six GRS at predetermined *p* < 0.05 in main analyses. In addition, we presented the results on GRS at all *p*-value thresholds as sensitivity analyses (Supplementary Table 1).

### Outlier analyses

We performed outlier analyses to detect influential cases that may have biased the regression models. These analyses were performed on the GRS *p* < 0.05 models (controlling for PC scores, age, and sex) with which significant or near-significant (*p* < 0.10) main or interaction effects were obtained. For this, we used the influence.ME package [55] to calculate Cook’s distance for all observations (i.e., one MADRS-S rating) and all individuals (i.e., all MADRS-S ratings by one individual). Possible influential observations and individuals were identified by visual inspection of the Cook’s distance plots, and the regression analyses were rerun with the outlying observations or individuals removed. Removing influential observations or individuals did not result in altered interpretations of the significant or near-significant results in any of the cases.

## Results

### Treatment effects

We observed a significant negative effect of time (B = −1.29, *p* < 2 × 10^−16^) and a significant positive effect of quadratic time (B = 0.048, *p* < 2 × 10^−16^) on MADRS-S ratings. See Supplementary Table 2 for mean weekly ratings on the MADRS-S for the whole group. These results indicated that the patients’ depression scores decreased during treatment, with larger declines in the beginning.

### Effects of GRS on treatment response

**Fig. 1:**
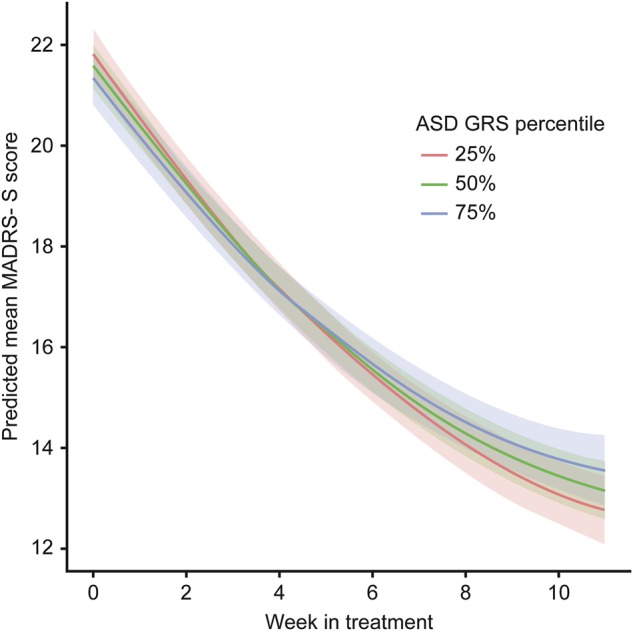
Effects of ASD GRS (at a *p*-value threshold of 0.05) on MADRS-S scores during iCBT treatment. The figure shows the predicted MADRS-S score for every week during treatment for three different levels of the ASD GRS (25th, 50th, and 75th percentiles). The shaded areas show the 95% confidence intervals of the predicted values. The participants with the highest ASD GRS scores (blue) showed poorer responses to treatment vs. those with average (green) or low (red) ASD GRS scores. Abbreviations: autism spectrum disorder (ASD), genetic risk score (GRS), Montgomery Åsberg Depression rating scale-Self (MADRS-S), internet-delivered cognitive behavior therapy (iCBT)

The first step in our analyses (GRS with *p* < 0.05 scores while controlling for ancestry PC scores) yielded one significant result. Namely, the ASD GRS was associated with MADRS-S changes over treatment time. This was reflected in the significant interaction effect (B = 0.09, *p* < .001) between ASD GRS and time (GRS × time): meaning that the higher the ASD genetic load, the less iCBT treatment response over time (Supplementary Table 1 and Fig. 1). This result remained significant after correcting for multiple testing. None of the other individual traits that were examined had a GRS with consistent significant effects on the MADRS-S scores or changes in MADRS-S scores over time.

## Discussion

This is the first study to explore the utility of GRS for predicting response to psychological treatment for patients with MDD. We set out to test the hypothesis that the GRS for six psychiatric and cognitive traits would be associated with treatment outcome. Participants with the highest ASD GRS showed a poorer response to treatment versus those with average or low ASD GRS.

The finding that ASD GRS was associated with outcome suggested that autism spectrum related genetic risk also puts one at risk for failing to respond to cognitive behavioral treatment for depression. If we assume that a high ASD GRS is associated with greater expression of autism spectrum phenotypes [56], we can speculate why ASD traits are related to worse MDD treatment outcome. For example, patients with high ASD GRS may have depressive symptoms due to ASD-related difficulties, such as problems with social communication and interaction, rather than phenotypes associated with MDD, such as distortive negative current thoughts and lack of reinforcing behavior. Consequently, iCBT for MDD would not target the main reasons for the depressive state in patients with higher load for ASD GRS, which could lead to even more negative emotions and feelings of failure. Furthermore, perhaps patients with a high ASD GRS load may have greater difficulty identifying with the rationale of the psychoeducational part of the therapy and thereby increase the risk of poorer response to the treatment.

Notably, MDD GRS was not associated with treatment outcome, and there are multiple possible explanations. First, the cause of depression and the treatment of depression may involve different genetic factors. In other words, MDD GRS might be related to why a person gets MDD but not related to the treatment response of MDD. Second, because we excluded patients with severe MDD, we may have selected for overall lower MDD GRS values. Finally, the GRS training set may not have been large enough.

The strengths of this study include the largest yet sample size in a genetic study focused on psychological therapy treatment response for MDD. Because all patients were diagnosed using a structured interview by a psychiatrist or supervised resident doctor suggests that the MDD phenotype was reliable. The iCBT treatment allows for tight control of what treatment was delivered with minimal risk for therapist or patient drift away from the treatment protocol. In addition, the study participants completed well-validated outcome measures at 12 time points (including pre- and post-assessments), thus producing a large body of treatment outcome data. The limitations of the study include that this study was likely still too small to detect robust and reliable associations with treatment response on both the aggregate (GRS) and individual locus (SNP) levels. In addition, the therapeutic mechanisms of iCBT can differ from traditional CBT and hereby limit the generalization of the results.

Our finding of an association of ASD GRS with CBT outcome is the first significant finding using a genome-wide approach in the field of therapygenetics. These preliminary findings need to be replicated before firm conclusions can be drawn and the possibility of the finding being a false positive must be considered.

## Electronic supplementary material

Supplementary Figure 1

Supplementary Figure 2

Supplementary Figure 1 Legend

Supplementary Figure 2 Legend

Supplemental tables
