# Supplementary figures and images for: Genetics of response to cognitive behavior therapy in adults with major depression: a preliminary report
Source: Mol Psychiatry. 2018 Nov 8;24(4):484–90. doi: 10.1038/s41380-018-0289-9 (PMC6477793; doi:10.1038/s41380-018-0289-9)

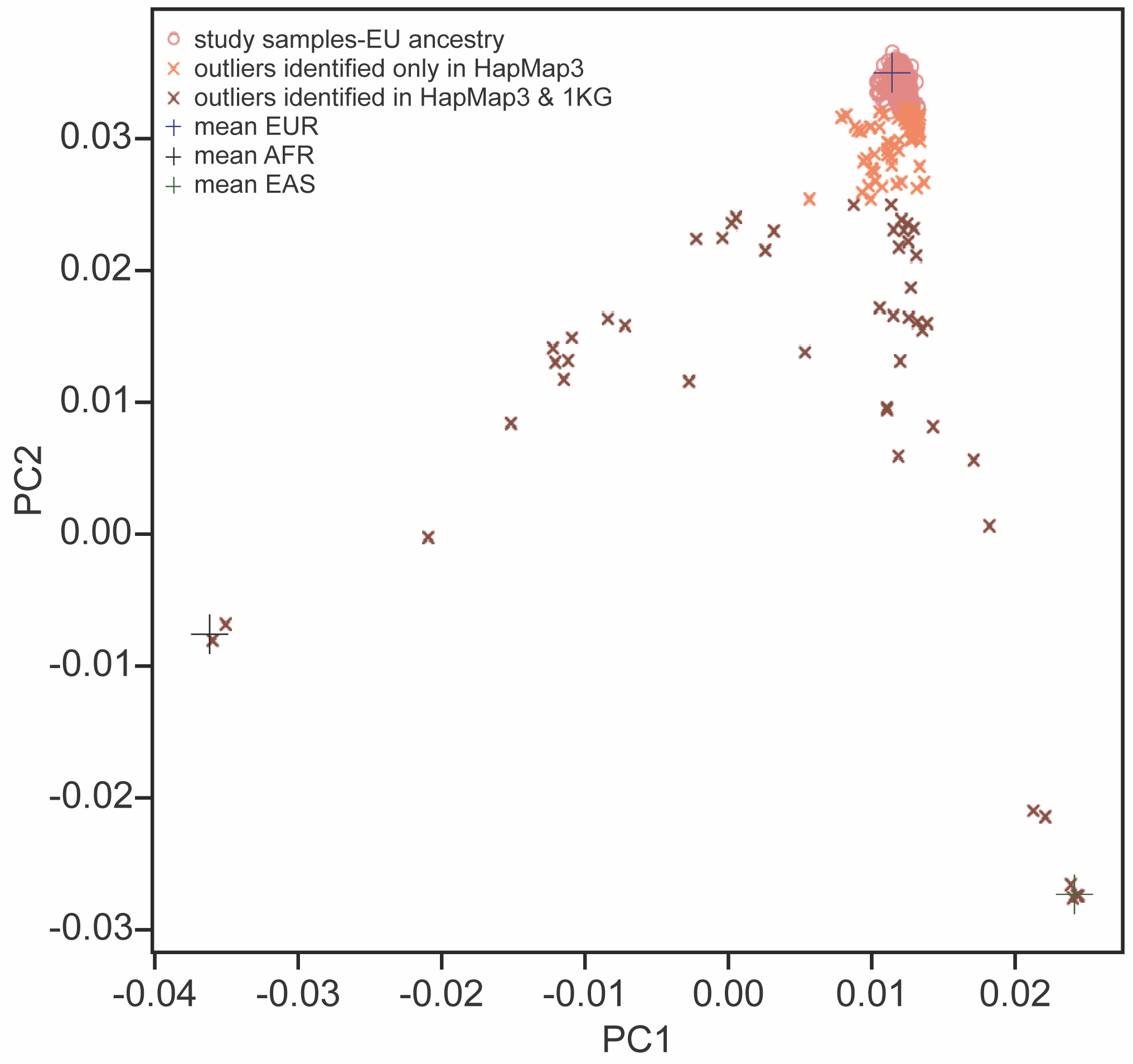

Supplement: Supplementary file 1 — Supplementary Figure 1 [file 41380_2018_289_MOESM1_ESM.tif]

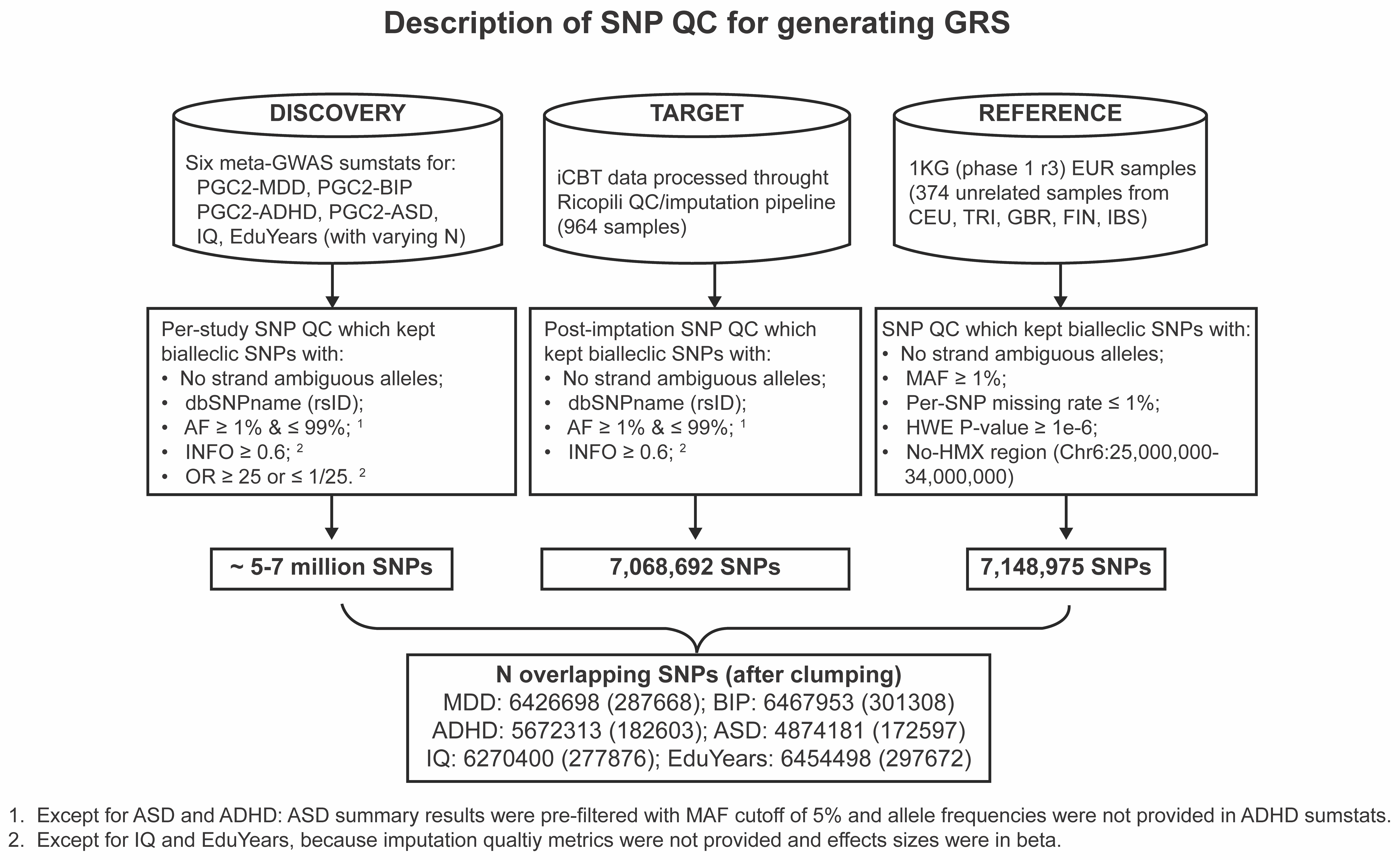

Supplement: Supplementary file 2 — Supplementary Figure 2 [file 41380_2018_289_MOESM2_ESM.tif]
