# Supplementary material for: Genetics of response to cognitive behavior therapy in adults with major depression: a preliminary report
Source: Mol Psychiatry. 2018 Nov 8;24(4):484–90. doi: 10.1038/s41380-018-0289-9 (PMC6477793; doi:10.1038/s41380-018-0289-9)
Supplement: Supplementary file 3 — Supplementary Figure 1 Legend [file 41380_2018_289_MOESM3_ESM.pdf]

Supplementary Figure 1. PCA of genotype data of 964 Swedish individuals who completed the iCBT trial. Forty-nine samples were excluded from the analysis based on being an outlier in both HapMap3 and 1KG. Abbreviations: principal component analysis (PC), European ancestry (EUR), African ancestry (AFR), east Asian ancestry (EAS)
