# Supplementary material for: Genetics of response to cognitive behavior therapy in adults with major depression: a preliminary report
Source: Mol Psychiatry. 2018 Nov 8;24(4):484–90. doi: 10.1038/s41380-018-0289-9 (PMC6477793; doi:10.1038/s41380-018-0289-9)
Supplement: Supplementary file 4 — Supplementary Figure 2 Legend [file 41380_2018_289_MOESM4_ESM.pdf]

Supplementary Figure 2. GRS calculation. Flow chart of the SNP QC of the discovery sets, target set, and reference data, together with the overlapping numbers of SNPs among these three sets. Abbreviations: single-nucleotide polymorphism (SNP), quality control (QC), genetic risk score (GRS), genome-wide association study (GWAS), allele frequency (AF), odds ratio (OR), 1000 Genomes (IKG), major depressive disorder (MDD), bipolar disorder (BIP), attention-deficit/hyperactivity disorder (ADHD), autism spectrum disorder (ASD), intelligence (IQ), educational attainment (EDU)
