# Supplementary material for: Genetics of response to cognitive behavior therapy in adults with major depression: a preliminary report
Source: Mol Psychiatry. 2018 Nov 8;24(4):484–90. doi: 10.1038/s41380-018-0289-9 (PMC6477793; doi:10.1038/s41380-018-0289-9)
Supplement: Supplementary file 5 — Supplemental tables [file 41380_2018_289_MOESM5_ESM.docx]

**SUPPLEMENTARY INFORMATION**

Supplementary Figure 1.

Supplementary Figure 2.

Supplementary Table 1.

# GRS_*p*- level

**Main effect Beta1**

**Main effect *p*- value**

**Interaction with time effect Beta1**

**Interaction with time**

**effect *p*- value**

| ASD_10-5 | -0.163  -0.082  0.093 | 4.50x10-1  7.00x10-1  6.66x10-1 | -0.002  0.002  0.023 | 9.30x10-1  9.22x10-1  3.39x10-1 |
| --- | --- | --- | --- | --- |
| ASD_10-4  ASD_0.001 |  |  |  |  |

| ASD_0.01 | -0.325 1.31x10-1 | 0.069 | **4.59x10-3** |
| --- | --- | --- | --- |
| ASD_0.05 | -0.405 5.72x10-2 | 0.091 | **1.46x10-4** |
| ASD_0.1 | -0.624 **3.40x10-3** | 0.085 | **4.43x10-4** |
| ASD_0.5 | -0.640 **3.10x10-3** | 0.083 | **7.07x10-4** |
| ASD_1.0 | -0.654 **2.52x10-3** | 0.084 | **6.43x10-4** |

**Supplementary Table 1.** Estimated effects of ASD on iCBT treatment response. The left column lists each GRS and the iCBT response measured by the Montgomery Åsberg Depression Rating Scale-Self at eight different *p*-level thresholds. Significant *p* values are in bold font. Abbreviations: genetic risk score (GRS), autism spectrum disorder (ASD), internet-delivered cognitive behavior therapy (iCBT)

Supplementary Table 2.

| **MADRS-S** | **N** | **Mean** | **SD** |
| --- | --- | --- | --- |
| MADRS-S  Baseline | 894 | 22.2 | 6.3 |
| week 1 | 817 | 19.7 | 6.6 |
| week 2 | 801 | 18.6 | 7.2 |
| week 3 | 767 | 17.4 | 7.6 |
| week 4 | 740 | 16.7 | 7.4 |
| week 5 | 733 | 16.5 | 7.4 |
| week 6 | 717 | 15.5 | 7.8 |
| week 7 | 676 | 14.5 | 7.7 |
| week 8 | 658 | 13.8 | 7.7 |
| week 9 | 642 | 13.0 | 7.5 |
| week 10 | 612 | 12.3 | 7.3 |
| MADRS-S Post | 789 | 12.8 | 7.9 |

**Supplementary Table 2.** Weekly MADRS-S measures. The table shows the N and mean and SD of the MADRS-S measures for the 12 assessment points. MADRS-S Baseline represents the first assessment, which was performed at the start of treatment, and the subsequent time points are the weekly assessments. MADRS-S Post represents the last assessment, which was performed

immediately after the treatment ended. Abbreviations: Montgomery Åsberg Depression rating scale-Self (MADRS-S), standard deviation (SD)
